# Interannual Variability of *Dinophysis acuminata* and *Protoceratium reticulatum* in a Chilean Fjord: Insights from the Realized Niche Analysis

**DOI:** 10.3390/toxins11010019

**Published:** 2019-01-05

**Authors:** Catharina Alves-de-Souza, José Luis Iriarte, Jorge I. Mardones

**Affiliations:** 1 Algal Resources Collection, MARBIONC, University of North Carolina Wilmington, 5600 Marvin Moss K. Lane, Wilmington, NC 29409, USA; 2 Instituto de Acuicultura and Centro de Investigación Dinámica de Ecosistemas Marinos de Altas Latitudes—IDEAL, Universidad Austral de Chile, Puerto Montt 5480000, Chile; jiriarte@uach.cl; 3 COPAS-Sur Austral, Centro de Investigación Oceanográfica en el Pacífico Sur-Oriental (COPAS), Universidad de Concepción, Concepción 4030000, Chile; 4 Instituto de Fomento Pesquero (IFOP), Centro de Estudios de Algas Nocivas (CREAN), Padre Harter 574, Puerto Montt 5501679, Chile; jorge.mardones@ifop.cl

**Keywords:** *Dinophysis acuminata*, *Protoceratium reticulatum*, Reloncaví Fjord, OMI analysis, WitOMI analysis, *Mesodinium* cf. *rubrum*, El Niño Southern Oscillation, Southern Annual Mode

## Abstract

Here, we present the interannual distribution of *Dinophysis acuminata* and *Protoceratium reticulatum* over a 10-year period in the Reloncaví Fjord, a highly stratified fjord in southern Chile. A realized subniche approach based on the Within Outlying Mean Index (WitOMI) was used to decompose the species’ realized niche into realized subniches (found within subsets of environmental conditions). The interannual distribution of both *D. acuminata* and *P. reticulatum* summer blooms was strongly influenced by climatological regional events, i.e., El Niño Southern Oscillation (ENSO) and the Southern Annual Mode (SAM). The two species showed distinct niche preferences, with blooms of *D. acuminata* occurring under La Niña conditions (cold years) and low river streamflow whereas *P. reticulatum* blooms were observed in years of El Niño conditions and positive SAM phase. The biological constraint exerted on the species was further estimated based on the difference between the existing fundamental subniche and the realized subniche. The observed patterns suggested that *D. acuminata* was subject to strong biological constraint during the studied period, probably as a result of low cell densities of its putative prey (the mixotrophic ciliate *Mesodinium* cf. *rubrum*) usually observed in the studied area.

## 1. Introduction

Diarrhetic Shellfish Poisoning (DSP) is a gastrointestinal syndrome caused by the consumption of shellfish contaminated with okadaic acid (OA) and dinophysistoxins (DTXs) produced by certain dinoflagellates of the genus *Dinophysis* and, to a lesser extent, by benthic *Prorocentrum* species [1]. DSP outbreaks caused by *Dinophysis* spp. have been mainly reported from temperate areas with well-developed aquaculture activities, mostly in Europe, Japan, and Chile [2]. Although only OA and DTXs have been linked to DSP [3], other lipophilic toxins (LSTs) such as pectenotoxins (PTXs) and yessotoxins (YTXs) are also included in seafood safety regulations because they are toxic to mice following intraperitoneal injection of lipophilic shellfish extracts, and, in the case of PTXs, have been shown to promote tumor formation in mammals [4]. PTXs production have been linked only to *Dinophysis* species while YTXs are known to be produced by the dinoflagellates *Protoceratium reticulatum*, *Lingulodinium polyedrum*, *Gonyaulax spinifera* and *G. taylorii* [4,5,6]. Azaspiracids (AZAs), produced by dinoflagellates of the genus *Azadinium* [7], have diarrheagenic effect on humans and are included in the European Union (EU) seafood safety regulations [4].

*D. acuta* and *D. acuminata* are the most frequent and abundant *Dinophysis* species in southern Chile’s fjords (53–41° S) [8,9,10,11,12,13]. DSP have been of special concern in this geographical area since the 1970s, when intoxications by diarrhetic toxins were first reported following the consumption of contaminated shellfish extracted from the Reloncaví Sound [14]. DTX-1 and DTX-3 are the predominant DSP toxins in southern Chile [15,16,17]. The chronicle occurrence of these toxins in bivalves from this area during spring–summer is usually associated with *D. acuta* [18] and less frequently with *D. acuminata* [9,19]. DTX-1 has been detected in plankton samples from this region [20,21], although the causative organism remains to be identified. More recently, DTX-2 has been detected in the plankton associated with the presence of *D. acuta* [13]. PTXs presence in southern Chile have been detected in filter feeders [22], plankton assemblages [13,20], and Diaion^®^ resin passive samplers [23], with the production of PTX-2 by *D. acuminata* confirmed in isolates from this area [24]. Finally, YTXs have been recorded in southern Chile both in bivalves and plankton samples containing *P. reticulatum* [12,21,25,26], whereas AZAs have been detected only in bivalves [27].

Despite the evident impact of DSP events in southern Chile, few field studies have focused on the ecological characterization of *Dinophysis* spp. in this area [8,10,21,28]. The available evidence from seasonal surveys points to the importance of persistent saline stratification and increased temperature to high cell densities of *D. acuminata* during spring–summer in the inner portion of fjords [28], where they have been observed forming thin layers associated with the pycnocline [21]. However, these findings were based on seasonal studies carried out over only 1–2 years without considering inter-annual environmental variability. On the other hand, information on *P. reticulatum* is especially scarce and restricted to an apparent preference of this species by high temperatures due to its occurrence during summer in southern Chilean fjords [21]. Although YTXs are not linked to DSP intoxications, moderate levels of these toxins under the EU regulation (1 mg K^−1^; [5]) have been linked to false positives in DSP mouse bioassays in southern Chile [21] which can lead to the unnecessary closure of areas to shellfish extraction. Thus, *P. reticulatum* distribution should also be determined when assessing the environmental conditions promoting the development of *Dinophysis* spp. and the conditions leading to high DSP toxicity in bivalves in southern Chile.

Here, we present the interannual distribution of *Dinophysis* spp. and *P. reticulatum* from May 2006 to February 2017 in a highly stratified estuarine system in southern Chile, the Reloncaví Fjord (~41.6° S). Our main goal was to obtain insight on the environmental conditions accounting for differences between years where *D. acuminata* and *P. reticulatum* blooms were observed and the ones without blooms of these species. For that, environmental conditions affecting spatio-temporal distribution of the two species over the 10-year time series were determined following a niche approach based on the Outlying Mean Index (OMI) [29]. Then, the Within Outlying Mean Index (WitOMI) [30,31] was used to decompose the species’ realized niche into realized subniches (found within subsets of environmental conditions) to estimate the impact of biological constraints on *D. acuminata* and *P*. *reticulatum* populations. Further WitOMI analyses for *D. acuminata* considering a complementary dataset (spring–summer 2008/2009) were performed, for which additional data on nutrient data and density of the ciliate *Mesodinium* cf. *rubrum* (the putative *Dinophysis* prey, [32]) were available.

## 2. Results

### 2.1. Physical and Meteorological Conditions

**Figure 1:**
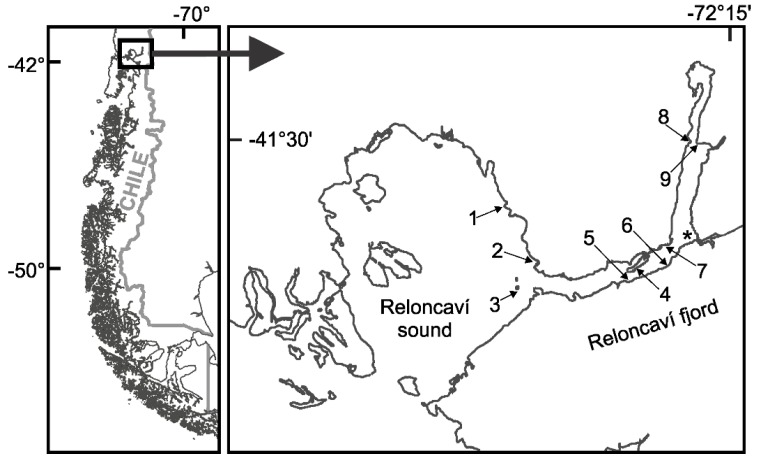
Location of the nine sampling stations in the Reloncaví Fjord. The asterisk indicates the position of the Puelo River’s inflow in the fjord.

**Figure 2:**
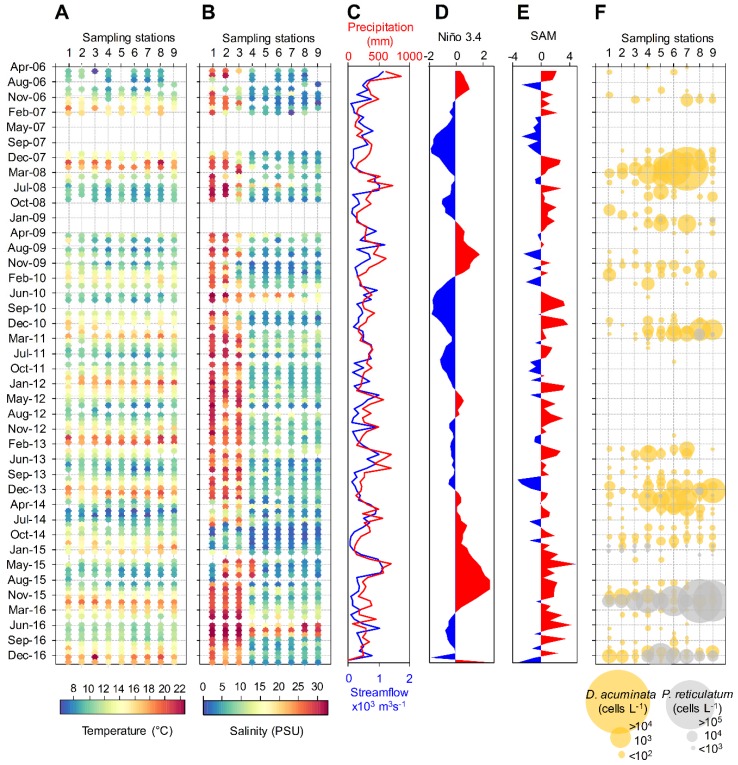
Spatio-temporal variability of physical conditions of the water column, meteorological conditions and potentially toxic dinoflagellate species in the Reloncaví Fjord for the 10-year time series. (**A**) subsurface water temperature, (**B**) subsurface salinity, (**C**) precipitation and streamflow from the Puelo River, (**D**) Niño 3.4 index, (**E**) Marshall Southern Annular Mode index (SAM), (**F**) cell density of *Dinophysis acuminata* and *Protoceratium reticulatum*.

The 10-year time series (May 2006 to February 2017) was obtained as part of a harmful algae monitoring program carried out by the Chilean Fishing Promotion Institute (IFOP; “Instituto de Fomento Pesquero”) at nine sampling stations (Figure 1): three in the Reloncaví Sound near to the mouth of the fjord (stations 1 to 3), four in the middle (stations 4 to 7) and two at the head (stations 8 to 9) of the fjord. The Reloncaví Fjord was characterized by strong spatio-temporal environmental heterogeneity regarding water temperature and salinity. Average maximal and minimal subsurface (depth ~ 1 m) water temperatures were 19 ± 2.2 °C and 8.2 ± 2.3 °C, respectively, with similar values observed among the different sampling stations in each season of the year (Figure 2A and Figure S1A). Maximal subsurface water temperatures were observed in January 2008 (22 °C) and January 2017 (22.6 °C) (Southern Hemisphere summer) while the lowest absolute value was observed in May 2006 (6.2 ± 2.2 °C). Subsurface salinity showed extreme oscillations (0.77–32.54 PSU) in all sampling stations throughout the study period (Figure 2B). Although no consistent seasonal pattern was observed regarding subsurface salinity values (Figure S1B), a clear spatial gradient was observed for this variable with lower values (<15 PSU) mostly observed in the inner part of the fjord (sampling stations 4 to 9).

Salinity was strongly stratified in the upper surface layer for the majority of the studied period. This vertical structure was due to significant differences in salinity between fresher near-surface waters (<15 PSU) and saltier subsurface marine waters (>30 PSU) (Figure S2). Less pronounced stratification was rarely observed and only during winter months, when higher salinities were occasionally observed in the upper layer. The Brunt–Väisälä buoyancy frequency (N_BV_), a proxy of the water column stratification [33], oscillated between 0.001 s^−1^ (homogeneous water column) to 0.15 s^−1^ (stratification with a sharp pycnocline), although stratification with a more gradual pycnocline was more frequently observed (N_BV_ ~ 0.025 s^−1^) (Figure S1C,I). The pycnocline depth oscillated between 2 and 9 m (Figure S1D). Besides temperature, seasonal variability was also related to precipitation with this variable strongly correlated to streamflow from the Puelo River (R = 0.58; *p* < 0.05). Higher values for both variables were mostly observed during winter months, although a second precipitation peak was observed during spring for some years (Figure 2C and Figure S1E,F). Interannual variability was related to oscillation in the El Niño Southern Oscillation (ENSO) (here accessed through the Niño 3.4 index) and the Southern Annual Mode (SAM) (Figure 2D,E and Figure S1G,H).

### 2.2. Spatio-Temporal Distribution of Dinophysis spp. and P. reticulatum

Five *Dinophysis* species were identified in the Reloncaví Fjord during the 10-year time series. *D. acuminata* was the most frequent species (present in 26% of the samples), followed by *D. punctata* (2% of the samples). *D. acuta*, *D. caudata* and *D. tripos* were less frequently observed (<1% of the samples). Other unidentified species were also observed and jointly quantified as *Dinophysis* spp. (present in only 5% of the samples). During the study period, *D. acuminata* was the only *Dinophysis* species with cell densities higher than the considered as bloom level for species of this genus (i.e., cell densities > 1000 cells L^−1^ [34]) Although *D. acuminata* was observed in all sampling stations, high cell densities of this species (>1000 cells L^−1^) were usually present in the inner portion of the fjord during the spring–summer months (October to March). Blooms of *D. acuminata* were observed in the years 2008 (11,300 cells L^−1^), 2011 (2800 cells L^−1^) and 2014 (4200 cells L^−1^), mostly during summer months (Figure 2F and Figure S3A). An exception was observed in the spring of 2008 when a bloom of this species was observed in the head of the fjord (2500 cells L^−1^). High cell densities (>1000 cells L^−1^) were also occasionally observed for this species during autumn and winter months. Although moderate densities of *P. reticulatum* (>1000 cells L^−1^) were occasionally observed throughout the study, blooms of this species were observed in 2016 (175,700 cells L^−1^) and 2017 (62,600 cells L^−1^), always during the summer (Figure 2F and Figure S3B).

### 2.3. Niche Analysis

**Figure 3:**
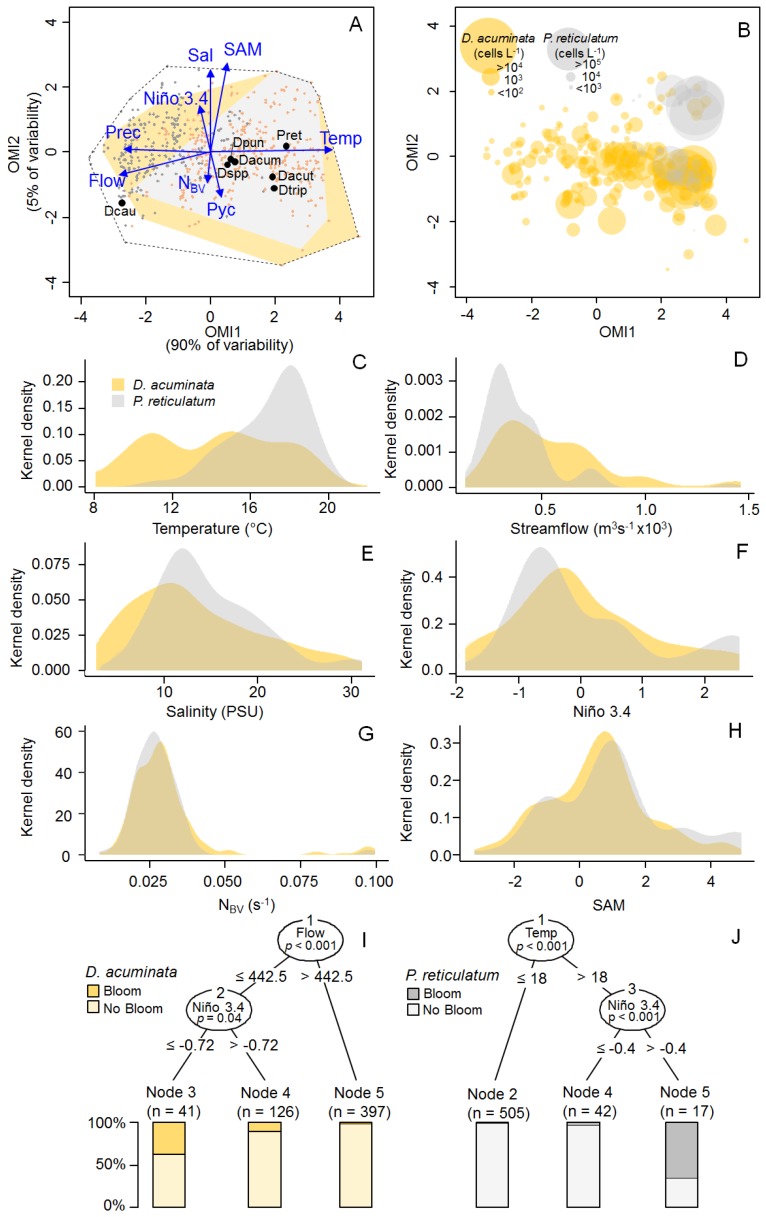
(**A**) Outlying Mean Index (OMI) analysis of the five *Dinophysis* species and *P. reticulatum* for the entire sampling period in the inner portion of the fjord (sampling stations 4 to 9). Blue vectors show relationship with the physical and meteorological variables. Samples from the spring–summer and autumn–winter periods are depicted in orange and grey, respectively. The dashed line delimitates the realized environmental space (i.e., sampling domain) whereas the yellow and grey polygons represent the realized niches of *D. acuminata* and *P. reticulatum*, respectively. The black dots represent the mean habitat condition used by the different species (i.e., species’ niche positions). Dacum = *D. acuminata*, Dacut = *D. acuta*, Dcau = *D. caudata*, Dpun = *D. puncata*, Dtrip = *D. tripos*, Pret = *P. reticulatum*; Flow = Puelo River’s Streamflow, Niño 3.4 = Niño 3.4 index, N_BV_ = Brunt–Väisälä buoyancy frequency, Pyc = depth of the pycnocline, Prec = precipitation, Sal = subsurface salinity, Temp = subsurface water temperature, SAM = Marshall Southern Annular Mode index. (**B**) Distribution of cell densities of *D. acuminata* and *P. reticulatum* in the OMI multivariate space. (**C**–**H**) Kernel density estimation (KDE) plots showing the frequency of occurrence (presence/absence) of *D. acuminata* and *P. reticulatum* related to different environmental variables. (**I**,**J**) Conditional inference trees showing main variables associated with blooms of *D. acuminata* (**I**) and *P. reticulatum* (**J**).

The Outlying Mean Index (OMI) was used to determine the combination of environmental variables that maximized average species marginality (i.e., the Euclidean distance between the mean habitat condition used by the species and the mean habitat condition of the sampling space [29]). A preliminary analysis using all samples (*n* = 839; not shown) indicated a strong spatial gradient due to differences in subsurface salinity observed between stations located in the exterior and the inner part of the fjord (Figure 2B). To remove the effect due to the spatial variability, only data for sampling stations 4 to 9 were included in a posterior analysis (*n* = 564). The OMI analysis considering only the samples from the inner part of the fjord depicted environmental gradients related to both seasonal and interannual temporal scales (Figure 3A). Together, the first two OMI axes explained 95% of the total explained variability. The OMI axis 1 accounted for the seasonal variability with the spring–summer period positively related to subsurface water temperature and negatively related to both precipitation and streamflow from the Puelo River, whereas the OMI axis 2 accounted for the interannual variability related mainly to the SAM and Niño 3.4 indexes as well as subsurface salinity. The *envfit* test [35] pointed out temperature, streamflow, SAM index as the variables accounting for most of total explained variability (R^2^ = 0.88, 0.55 and 0.33, respectively; *p* < 0.01).

The OMI (i.e., species marginality) depends on the deviation from a theoretical ubiquitous, uniformly distributed species that would occur under all available habitat conditions (i.e., observed in all samples) (OMI = 0) and is inversely related to the tolerance index (an estimate of niche breath) [29]. Species with low OMI occur in typical (or common) habitats of the sampling region. They usually show high tolerance and are associated with a wide range of environmental conditions (i.e., generalists). On the contrary, species with high OMI occur in atypical habitats and are expected to have low tolerance associated with a distribution across a limited range of environmental conditions (i.e., specialists). From the six species included in the analysis, *D. acuminata*, *D. caudata*, *D. tripos,* and *P. reticulatum* showed significant OMIs (*p* < 0.05). The most uniformly distributed species was *D. acuminata* (OMI = 0.6) (used typical habitat), whereas *D. caudata* was the most specialized species followed by *D. tripos* and *P. reticulatum* (OMI = 18.80, 5.84 and 5.75, respectively) (using more atypical habitat) (Table S1).

In the OMI multivariate space, the polygon formed by all samples corresponded to the “realized environmental space” whereas the polygon formed only by the samples where a given species is present correspond do the “realized niche” of the species [36]. Both *D. acuminata* and *P. reticulatum* occupied large portions of the realized environmental space (Figure 3A,B), with the later showing a comparatively narrower realized niche (tolerance = 3.26 and 1.07, respectively). *D. acuminata* and *P. reticulatum* showed high residual tolerance when compared to the other species (8.77 and 8.38, respectively) and for both species this niche parameter accounted for more than 50% of their variability (which indicates that most variability in the niche of the two species was not explained by the environmental variables included in the analysis).

Kernel density estimation (KDE) plots obtained separately for each variable (Figure 3C–H) revealed some patterns regarding the presence/absence of these two species and environmental conditions. Based on that, *D. acuminata* were more frequently observed in conditions with salinities lower than 15 PSU, whereas *P. reticulatum* showed preference for temperatures between 16 and 18 °C and salinity between 10 and 15 PSU. Both species were related to Puelo River’s streamflow lower than 1000 m^3^ s^−1^, negative to slightly positive values of the Niño 3.4 index, slightly positive values of the SAM index and N_BV_ values of ~0.025 s^−1^ (which was indicative of stratified conditions with a gradual pycnocline). Conditional inference trees indicated streamflow lower than 500 m^3^ s^−1^ associated with negative values of the Niño 3.4 index (<−0.4) as the conditions leading to *D. acuminata* blooms (Figure 3I). On the other hand, blooms of *P. reticulatum* occurred under temperatures higher than 18 °C and values of the Niño 3.4 index higher than −0.7 (Figure 3J).

### 2.4. Subniche Analysis

The Within Outlying Mean Index (WitOMI) [30] was used to decompose the ecological niche of the different species into subniches (i.e., subset of habitat conditions used by a species) taking into account interannual subsets of samples. This analysis is a refinement of the OMI analysis and provides estimations of niche shifts under different subsets of habitat conditions [31]. Considering that temperature was the main factor determining the distribution of the species in the OMI analysis when taking into account all samples and that dinoflagellate blooms were observed mostly during summer months, we decide to perform the subniche analysis considering only the samples for this season of the year (*n* = 167) to remove the effect of seasonal variability. As we aimed to detect the main conditions leading to the formation of blooms, only moderate to high cell densities for these two species were considered (≥1000 and ≥10,000 cells L^−1^ for *D. acuminata* and *P. reticulatum*, respectively).

#### 2.4.1. Subsets

Summer samples from a given year were classified according to the occurrence/absence of blooms of different dinoflagellate species in the Reloncaví Fjord. Although the dinoflagellate *Prorocentrum micans* was not considered in this study, a massive bloom of this species was observed in March 2009 [37]. Thus, summer samples of this year were considered as a separate subset. According to this criterion, four subsets were recognized: (1) summer samples of years with *D. acuminata* blooms (2008, 2011, and 2014), (2) summer samples of year 2009 for which the massive *P. micans* bloom was observed, (3) summer samples of years where *P. reticulatum* blooms were observed (2015, 2016 and 2017), and (4) summer samples of years where no dinoflagellate bloom was observed.

**Figure 4:**
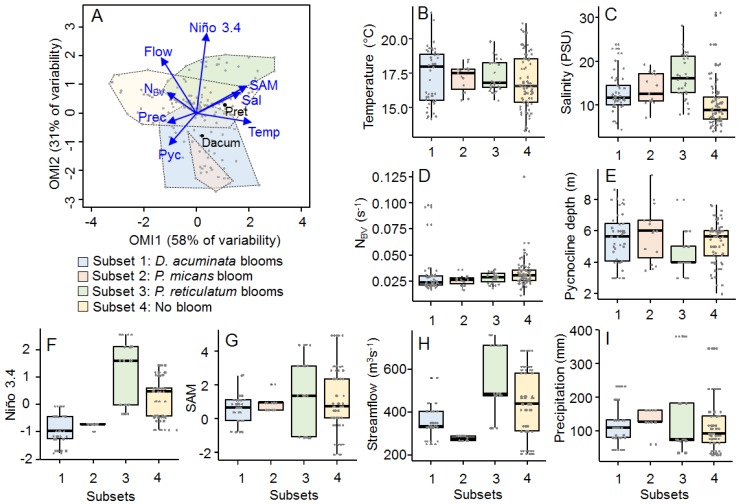
(**A**) Distribution of the four subsets in the OMI multivariate space considering only the summer period in the inner portion of the Reloncaví Fjord (sampling stations 4 to 9) and their relationship with environmental variables (blue vectors; see Figure 3 for meaning of labels). (**B**–**I**) Boxplots showing differences in the four subsets regarding subsurface water temperature (**B**), subsurface salinity (**C**), N_BV_ (Brunt–Väisälä buoyancy frequency) (**D**), pycnocline depth (**E**), Niño 3.4 index (F), SAM index (**G**), Puelo River’s streamflow (**H**) and precipitation (**I**). Horizontal lines indicate the median for the different variables.

The first two axes of the OMI analysis considering only samples from the summer period explained 89% of the total variability (Figure 4A). The OMI axis 1 accounted for the environmental temporal variability within the summer period and it was positively related to subsurface water temperature, subsurface salinity and SAM index and negatively related to precipitation and N_BV_. The OMI axis 2 accounted for the interannual variability and was positively related to the Niño 3.4 index and streamflow. The *envfit* test indicated the Niño 3.4 index, streamflow and the SAM index as the main variables accounting for the total explained variability (R^2^ = 0.62, 0.43 and 0.36, respectively; *p* < 0.01).

The four recognized subsets were distributed along the OMI axis 2 (Figure 4A). The function *subkrandtest* (implemented in the package ‘subniche’ in R) indicated that the main differences among the four subsets were given by the Niño 3.4 index, Puelo River’s streamflow, depth of the pycnocline and subsurface salinity whereas no significant differences were observed regarding subsurface temperature, N_BV_ and the SAM index (Figure 4B–G; *p* > 0.001). Subset 1 (summers from years where *D. acuminata* blooms were observed) was characterized by low streamflow and negative values of the Niño 3.4 index. These conditions were also observed for subset 2 (summer months from the year where the massive bloom of *P. micans* was observed; [37]). Subset 3 (summer samples from years where *P. reticulatum* blooms were observed) was characterized by high Puelo River’s streamflow, positive values of the Niño 3.4 index associated with slightly higher subsurface salinities and more superficial pycnocline when compared to the other subsets. Subset 4 (summers of years with no dinoflagellate blooms) was characterized by lower salinity and neutral values of the Niño 3.4 index. Although no significant difference was observed for the SAM index, this index showed a broader range in subsets 3 and 4, when compared to subsets 1 and 2.

**Figure 5:**
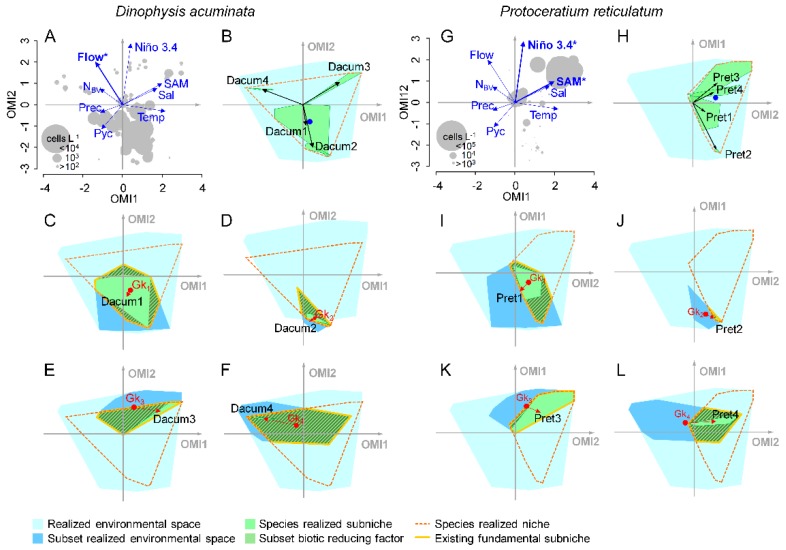
(**A**–**F**) *Dinophysis acuminata* and (**G**–**L**) *Protoceratium reticulatum* subniches’ dynamics considering only the summer period in the inner portion of the Reloncaví Fjord (sampling stations 4 to 9) and their relationship with the physical and meteorological variables (blue vectors; see Figure 3 for meaning of labels). Main variables associated with bloom conditions for both species (detected by conditional inference tree analyses) are indicated with asterisks. The blue dots represent the mean habitat condition used by the species in the entire sampling domain (i.e., species’ niche position) whereas the black labels represent the mean habitat condition used by the species in the subset (i.e., species’ subniche position). The black and red vectors represent species marginalities (i.e., WitOMI*G* and WitOMI*G_k_*, respectively). The red dots represent the mean habitat condition in each subset (*G_k_*). The light blue polygon represents the realized environmental space (i.e., sampling domain). For each species, the existing fundamental subniches (polygons delimited by yellow lines) are given by the overlap between the subsets (dark blue polygons) and the species’ realized niche (polygon delimited by dashed orange line). The difference between the existing fundamental subniche and the species subniche (light green polygons) is the “subset biotic reducing factor” (green area highlighted by diagonal lines), which correspond to the biological constraint exerted on the species that can be caused either by negative biological interactions or species dispersal limitations.

A clear separation between *D. acuminata* and *P. reticulatum* was shown by plotting their cell densities in the OMI multivariate ordination space of the summer period (Figure 5). As expected, the two species were related to the same conditions previously described as typical for the subsets where their blooms were observed (subsets 1 and 3, respectively) (Figure 5A,G). Additional conditional inference tree analysis, taking into account only the samples from the summer period, indicated that blooms of *D. acuminata* were mainly related to low streamflow whereas blooms of *P. reticulatum* were related to positive values of the Niño 3.4 and SAM indexes (not shown).

#### 2.4.2. Subniches

The WitOMI analysis allowed the calculation of two additional marginalities: the WitOMI*G* (i.e., Euclidean distance between the mean habitat condition used by the species in the subset and the mean habitat condition of the sampling domain) and the WitOMI*G_k_* (i.e., Euclidean distance between the mean habitat condition used by the species in the subset and the mean habitat condition of the subset). In ecological terms, the WitOMI*G* allows the detection of shifts in the mean habitat conditions used by the species in each subniche whereas the WitOMI*G_k_* represents the marginality of the species within the subset (i.e., if the species uses typical or atypical habitat in the subset) [30].

Based on this approach, we detected significant shifts in the subniche position of *D. acuminata* and *P*. *reticulatum* in the different subsets (Figure 5B,H). Although *D. acuminata* was distributed over the entire summer period, its used habitat was more marginal in the subsets 2, 3 and 4 (WitOMI*G* = 13.54, 6.40 and 10.54, respectively) than in subset 1 (WitOMI*G* = 1.74) (Table S2). This suggest that *D. acuminata* had a preference for the environmental habitat conditions in the subset 1. Furthermore, this species was significantly less marginal in subset 1 (WitOMI*G_k_* = 0.16; Figure 5C) when compared to subsets 2, 3 and 4 (WitOMI*G_k_* = 1.66, 2.66 and 5.56, respectively; Figure 5D–F). On the other hand, *P. reticulatum* used more common habitat in subset 1 and 3 (WitOMIG = 3.14 and 3.12, respectively) when compared to subsets 2 and 4 (WitOMIG = 12.34 and 5.44, respectively) (Table S2). Although, *P. reticulatum* showed similar marginality in the first two subsets, its realized subniche was comparatively broader in subset 3 than in subset 1 (tolerance = 1.08 and 0.24, respectively).

In the OMI multivariate space, the overlap between the polygon formed by samples of a subset and the polygon formed by the realized niche of a given species generates a third polygon that constitutes the “fundamental subniche” of this species. The area delimited by the difference between the fundamental subniche and the realized subniche correspond to the “subset biotic reducing factor”, i.e., biological constraint (*S_B_*) exerted on the species subniche that can be caused either by negative biological interactions or species dispersal limitations [30]. Both *D. acuminata* and *P. reticulatum* occupied a large position of their fundamental subniches in the subsets where their blooms were observed (subset 1 and 3, respectively) (Figure 5C,L).

**Figure 6:**
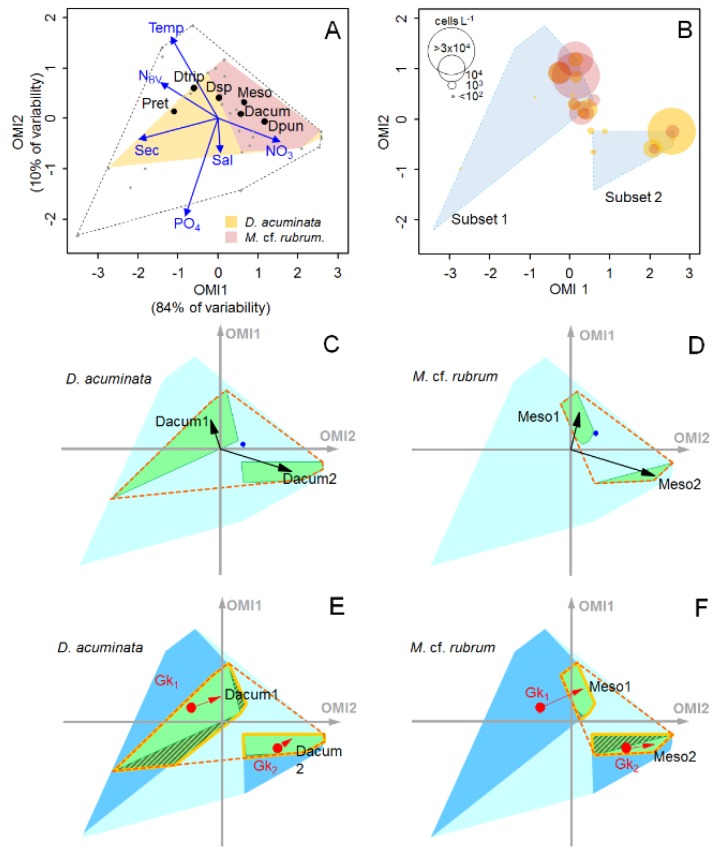
(**A**) OMI analysis for the summer–spring period 2008/2009 in the sampling station 8. The blue vectors show relationship with the physical and meteorological variables (see Figure 3 for meaning of labels). The black dashed line delimitates the realized environmental space (i.e., sampling domain) whereas the yellow and pink polygons represent the realize niches of *D. acuminata* and *M.* cf. *rubrum*, respectively. The black dots represent the mean habitat condition used by the different species (i.e., species’ niche positions). Dacum = *D. acuminata*, Dpun = *D. puncata*, Dtrip = *D. tripos*, Dsp = *Dinophysis* sp., Meso = *Mesodinium* cf. *rubrum*, Pret = *P. reticulatum*. (**B**) Distribution of cell densities of *D. acuminata* and *M.* cf. *rubrum* in the OMI multivariate space. Blue polygons represent the two subsets. (**C, E**) *D. acuminata* and (**D, F**) *M.* cf. *rubrum* subniches’ dynamics (see Figure 5 for meaning of dots, arrows and polygons).

To further assess the relative importance of biotic interactions on the subniche dynamics of *D. acuminata*, we performed an additional WitOMI analysis using a complementary dataset (previously published by Alves-de-Souza et al. [21]) that includes information on nutrient concentrations and the ciliate *M.* cf. *rubrum*. This sub-dataset was based on samples obtained every 2–3 weeks between October 2008 and March 2009 (spring–summer) from sampling station 8 (See Alves-de-Souza et al. [21] for a description of the environmental conditions during this period). The first two axes of the OMI analysis for this sub-dataset explained 94% of the variability. *D. acuminata* occupied common habitat (OMI = 0.35, tolerance = 1.69) when compared to *M.* cf. *rubrum* that was more marginal and showed a narrower realized niche (OMI = 0.52, tolerance = 0.45) (Figure 6A). Two subsets were stablished by k-mean cluster analysis of the OMI sample scores (see Methods). The OMI axis, positively related to the subset 2 (where *D. acuminata* bloom occurred), was mostly explained by NO_3_^−^ and Secchi disk (proxy of water transparency), whereas the OMI axis 2 was related to the subset 1 (higher *M.* cf. *rubrum* cell densities) and explained mainly by temperature and PO_4_^3−^ (Figure 6A,B). Although PO_4_^3−^ was the variable that accounted for most of the explained variability (*envfit*; R^2^ = 0.84), the species distribution along the OMI axis 1 seems to have been more related to Secchi disk, temperature and NO_3_^−^ (R^2^ = 0.79, 0.77, 0.50, respectively). Both *D. acuminata* and *M.* cf. *rubrum*. showed shifts in the subniche position and marginality (Figure 6C,D), with a stronger biotic constraint on *D. acuminata* in the realized subniche of the subset 1 (in samples where *M.* cf. *rubrum* was absent) (Figure 6E). Similarly, a strong biotic constraint on *M.* cf. *rubrum* was observed in the subset 2, concomitantly with *D. acuminata* blooms (Figure 6F).

## 3. Discussion

### 3.1. Seasonal and Interannual Variability 

*D. acuminata* and *P. reticulatum* are widespread HAB species observed in temperate areas worldwide [34,38]. High densities of these two species have been previously reported during summer months in southern Chilean fjords [9,21,28,39]. Although they are frequently observed in low densities (<100 cells L^−1^) [11,40,41], their blooms have being mostly regarded as episodic events of erratic occurrence. Here, we present for the first time evidence indicating that, far from having a random occurrence, blooms of both *D. acuminata* and *P. reticulatum* in the Reloncaví Fjord (and probably other fjord systems in southern Chile) are seasonal periodic events related to climatic and hydrological events of regional scale (i.e., ENSO, SAM). Moreover, our results showed that both species have different niche preferences that explain their seasonal and interannual distribution.

Results from the niche analysis considering the entire sampling period indicated that temperature and streamflow from the Puelo River were the main environmental factors associated with the seasonal variability in the Reloncaví Fjord (*envfit*; R^2^ = 0.88 and 0.55, respectively) (Figure 3A). The reduced freshwater streamflow values during summer months were in agreement with the historical trend reported for this fjord, characterized by a streamflow bimodal regime with two main peaks in winter and spring related to precipitation and snowmelt, respectively [42,43]. Although blooms of both *D. acuminat*a and *P. reticulatum* were associated with low streamflow (Figure 3D), the restricted occurrence of *P. reticulatum* during summer months seemed to be primarily determined by a preference of this species for surface water temperatures between 16 °C and 18 °C (Figure 3C), as previously reported for this species in culture experiments [44,45]. However, despite the fact that optimal temperatures were observed every summer, blooms of the species were only observed in years with values of the Niño 3.4 index higher than −0.4 (Figure 3J). *D. acuminata* presence was associated with a wide range of temperatures (Figure 3C) as usually observed for this species in other parts of the world [34]. These results were in disagreement with those previously reported for the Pitipalena Fjord (38°47′ S; 72°56′ W), where high temperature was suggested as a triggering factor for blooms of this species [28]. Instead, reduced streamflow from the Puelo River was found to be the main variable explaining high cell densities of *D. acuminata* in the Reloncaví Fjord during summer months, although blooms of this species were only observed in years with values of the Niño 3.4 index lower than −0.72 (Figure 3I).

The conditional inference trees suggest a hierarchical relevance of variables acting at different temporal scales in the formation of *D. acuminata* and *P. reticulatum* blooms. For both species, the first nodes of the trees were related to environmental conditions that were more relevant at the seasonal scale (i.e., low streamflow for *D. acuminata* and high temperature for *P. reticulatum*), whereas the second node depicted the main variable acting at the interannual scale (Niño 3.4 for both species) (Figure 3I,J). Shifts in the relative importance of environmental conditions according to the considered temporal scale have been previously reported for other microbial communities [46,47,48], with the interplay between factors acting at both seasonal and interannual scales determining the time-window of species occurrence [49]. In the specific case of the Reloncaví Fjord, our results indicate that although the time window for occurrence of *D. acuminata* and *P. reticulatum* are determined by variables acting at the seasonal scale, the formation of their blooms are ultimately defined by hydrological and climatological conditions acting at an interannual scale.

### 3.2. Summer Subsets

The niche and subniche analyses considering only the summer dataset allowed us to obtain a better understanding of the factors behind the interannual variability in blooms of *D. acuminata* and *P. reticulatum* during the 10-year time series (Figure 4 and Figure 5). These analyses indicated that the interannual distribution of the two species was also related to the SAM index in addition to the effect of streamflow and Niño 3.4 index (*envfit*; R^2^ = 0.36, 0.62 and 0.43, respectively; *p* < 0.01). Although reduced Puelo River’s streamflow is generally observed in summer months, when compared to winter and spring [42,43], summers with *D. acuminata* blooms (subset 1) were characterized by even lower values of streamflow than the typically observed for this season of the year (Figure 4H and Figure 5A) in association with the most negative values of the Niño 3.4 index (la Niña conditions) for the studied period (Figure 3F) (*subkrandtest*; *p* < 0.001 for both streamflow and Niño 3.4 indexes). On the other hand, summers with *P. reticulatum* blooms (subset 3) were characterized by positive values of the Niño 3.4 index (El Niño conditions) (*subkrandtest*; *p* < 0.001). This subset was further differentiated by higher variability in the SAM index, with a median value that was slightly more positive than the observed median values for the other subsets (Figure 4G). Although these differences were not significant (*subkrandtest*; *p* = 0.128), higher *P. reticulatum* cell densities were positively related to the SAM index (Figure 5G). This was further confirmed by a conditional inference tree considering only the summer dataset, which indicated positive values for both Niño 3.4 and SAM indexes as the main conditions associated with blooms of this species. These conditions were associated with higher salinities and shallower pycnoclines in the subset 3 (Figure 4C,E) (*subkrandtest*; *p* = 0.128).

ENSO is a coupled ocean-atmosphere phenomenon over the equatorial Pacific, characterized by irregular fluctuations between warm (El Niño) and cold (La Niña) conditions in the sea surface temperature, whereas SAM is an atmospheric mode of circulation that appears to modulate the air temperature over the southern tip of South America caused by pressure anomalies between the Antarctic and the 40–50° S circumpolar band [50]. The El Niño conditions are related to increase in the sea surface pressure (SLP) and weakening of westerlies in the southern extreme of the continent that may result in lower precipitation in western Patagonia when compared to average conditions [51]. At the same time, positive SAM levels lead to the intensification of the westerlies around the Antarctic periphery and weakening around 40° S, causing lower precipitation and increased air temperature over western Patagonia [43,52].

The relevance of the ENSO/SAM interplay for the occurrence of harmful algal blooms (HABs) in southern Chilean fjords has been demonstrate in a previous study, where it explained the formation of the most impressive HAB observed to date in southern Chile caused by the phytoflagellate *Pseudochatonella verruculosa* (Dictyochophyceae) in February–March 2016 [43]. As this bloom was observed just after the *P. reticulatum* bloom in January 2016 (the denser bloom of this species in the entire 10-year time series), both events were likely affected in a similar way by the existing climatological conditions. At this opportunity, Leon-Muñoz et al. [43] proposed that the combination between the strong El Niño event and the positive phase of SAM led to very dry conditions (both in terms of low precipitation and reduced freshwater input) associated with high radiation and reduced westerly wind, which in turn resulted in weakening of vertical stratification and the consequent advection of more saline and nutrient rich waters that ultimately determined the formation of the *P. verruculosa* bloom. Our data does not entirely support this hypothesis as (1) no significant difference among the different summer subsets was detected regarding either precipitation or the Brunt–Väisälä frequency (proxy of vertical stratification) and (2) the Puelo River’s streamflow levels for subset 3 (that includes the summer 2016) were actually higher when compared to the other summer subsets (*subkrandtest*; *p* > 0.001). This discrepancy could be explained by the differential treatment of the data as well as the considered time-window in both studies: while Leon-Muñoz et al. [43] detected a decreasing trend for both precipitation and river streamflow based on accumulated annual values of these variables in the last five decades, we based our conclusions on the comparison of monthly values only in the last decade. Similarly, analyses reinforcing our conclusions were based on hundreds of CTD profiles collected during throughout the study period. Although it is clear that a trend does exist regarding the decrease in precipitation and Puelo River’s streamflow in the Reloncaví Fjord [42,43], our results indicate that it was not the factor explaining the *P. verruculosa* and *P. reticulatum* blooms during the summer 2016. Instead, blooms of both species were explained by an increase in salinity (as suggested by Leon-Muñoz et al. [43]) likely caused by the shallowing of the pycnocline that facilitated the advection of more saline and (supposedly) nutrient rich water to the surface.

Of special notice was the occurrence of the massive bloom of the dinoflagellate *P. micans* in the summer 2009 (~10^5^ cells L^−1^; [37]), despite the fact that environmental and climatological conditions (low Puelo River’s streamflow and La Niña conditions) seemed to be favorable for *D. acuminata* blooms. This was interesting, as both species seem to have an overlap on their realized subniches. The reason for this discrepancy could be potentially related to variables that were not considered in the present study (e.g., dissolved inorganic nutrient concentrations). While both species are mixotrophic, they show very distinctive nutritional strategies: *P. micans* seems to be mostly facultative mixotroph, whereas *D. acuminata* relies on both photosynthesis and feeding on the ciliate *M.* cf. *rubrum* [34]. While *D. acuminata* shows high affinity by regenerate nitrogen sources (i.e., ammonia and urea) [53,54], a positive relationship between blooms of *M.* cf. *rubrum* and nitrate concentrations have been observed [55]. Available nutrient information for the head of the Reloncaví Fjord indicated that both nitrate and silicic acid concentrations were significantly lower in summer 2009 when compared to summer 2008 [21], which could have potentially favored the *P. micans* bloom formation.

The reduced river streamflow in the subsets 1 and 2 associated with negative values of Niño 3.4 index is intriguing, since higher precipitations could be expected during La Niña conditions [50]. The absence of significant differences in precipitation among the subsets could be explained by the lack of a clear signal in El Niño/La Niña conditions in the southern extreme of South America regarding precipitation [50]. While precipitation and streamflow were correlated (R = 0.58; *p* > 0.01) [42], no significant differences among the four subsets were detected regarding the former variable. This suggests that river streamflow levels during the study period depended mostly on snowmelt that could be expected to be less important in colder years (La Niña conditions). Another interesting aspect is the counter intuitive lack of correlation between streamflow levels and the depth of the pycnocline, with subsets 1 and 3 (with lower streamflow) showing deeper pycnoclines when compared to subset 3 (with higher streamflow). The explanation for this remains elusive, but could be related to microscale oceanographic aspects, such as internal waves or seiches [56].

### 3.3. Subniches and Reducing Biotic Factors

Several aspects revealed by the subniche analysis (Figure 5) further confirmed the preference of *D. acuminata* and *P. reticulatum* for the environmental conditions observed in the subsets where their blooms occurred (subsets 1 and 3, respectively). First, the broader realized subniches of the two species in their respective subsets (indicated by larger tolerances in Table S2) indicated a better efficiency in using the available resources [30]. This is also evidenced by a larger occupied portion of their fundamental subniches in these subsets (i.e., fundamental and realized niches have similar areas) (Figure 5C,L). The unfavorable conditions for *D. acuminata* and *P. reticulatum* in the years without dinoflagellate blooms (subset 4) are reflected in larger values of WitOMI*G* for both species in this subset (indicated by the length of the red arrows in Figure 5F,M).

One of the advantages of the WitOMI analysis is that it allows the estimation of the biological constraints (*S_B_*) exerted on the species, which is proportional to the space unoccupied by the species in its fundamental subniche (indicated by the green area highlighted by diagonal lines in Figure 5) [30]. This absence is interpreted as caused by biological constraint and can be due either to negative biotic interactions (e.g., parasitism, predation) or dispersal limitation of the species itself [57]. Interestingly, both *D. acuminata* and *P. reticulatum* showed a smaller unoccupied portion of their fundamental niches (attributed to biological constraint) in the subsets where their blooms were observed (Figure 6C,L, respectively). In the case of *D. acuminata*, the magnitude of biological constraint was even more impressive in the subset 4 (years without dinoflagellate blooms) (Figure 5F).

Although these results need to be interpreted with caution, the observed pattern shown in Figure 5C–F suggest that *D. acuminata* occurrence in the Reloncaví Fjord during the studied period was mostly modulated by biological constraints. Among the biotic factors affecting *Dinophysis* spp. dynamics, the availability of its prey (the ciliate *M.* cf. *rubrum*) is by far the most relevant [58]. Species of this genus are obligate mixotrophs that require both feeding on *M.* cf. *rubrum* and light for sustained growth [32,58,59,60]. In addition, M. cf. *rubrum* also depends on the ingestion of live prey to sustain growth (i.e., cryptophytes of the genera *Teleaulax* and *Geminigera*) [61]. In field populations, *Dinophysis* species may be under prey limitation for long periods, with maximal cell densities being preceded or co-occurring with high densities of *M.* cf. *rubrum* ciliates, resulting in predator–prey encounters and interactions [62,63,64], suggesting that the presence of the *Dinophysis*-*Mesodinium*-cryptophytes food chain may be used as a good indicator of upcoming *Dinophysis* spp. blooms [64].

*D. acuminata* blooms in co-occurrence with *M.* cf. *rubrum* was previously reported in the Reloncaví Fjord in a study using a sampling frequency of 2–3 weeks [21]. In this study, we revisited this dataset using the WitOMI approach to estimate the degree of biological constraint on both species during their period of co-occurrence. As the *S_B_* estimation is based on the absence of the species in sampling units where it should be present (as they are encompassed into the species fundamental niche), this approach can be extremely useful to obtain insights on biotic interactions involving time-lags (such as predator–prey interactions) even when short-frequency data are not available. Indeed, the clear mismatch between maximal cell densities of *D. acuminata* and *M.* cf. *rubrum* (Figure 6B) was congruent with the time-lagged correlation observed in other studies [62,63,64], whereas the strongest biological constraint on *D. acuminata* was observed in periods where *M*. cf. *rubrum* was absent (Figure 6E). Similarly, a strong biotic constraint on the ciliate was observed concomitantly to highest *D. acuminata* cell densities (Figure 6F).

### 3.4. Additional Aspects Affecting D. acuminata

Although *D. acuminata* is observed under a broad range of environmental conditions, blooms of this species are consistently associated with increased stratification [65]. Similarly, physical driving forces (e.g., wind and/or currents) causing accumulation/dispersion of *D. acuminata* cells have been pointed out as an important factor [66,67,68,69]. DSP events in bays used for shellfish production are frequently observed after the transport of *D. acuminata* cells from the near continental shelf [70,71,72], where they are frequently found in dense populations (>10^4^ cells L^−1^) occurring in thin layers [73,74]. Formation of *D. acuminata* blooms within coastal areas is better understood in upwelling influenced systems, more specifically the Galician rías [34]. In these systems, *D. acuminata* blooms are mostly initiated after the advection of cells from offshore (“pelagic seed banks”) with upwelled waters [62], although blooms can also be originated from persistence of autochthonous winter populations [75]. In both cases, accumulation of cells in the pycnocline and encounter with the mixotrophic ciliate *M.* cf. *rubrum* lead to cell proliferation [62,63].

By comparison, little is known about the environmental conditions leading to the development of *D. acuminata* blooms in fjords. The information from Swedish fjords points out to the formation of *D. acuminata* thin layers associated with strong stratification caused by freshwater input from land run-off, e.g., [76,77], whereas the interannual variability of this species seems to be related to climatic events of regional scale (i.e., the North Atlantic Oscillation; NAO) [78] favoring the entrainment and advection of cells from offshore [79]. Although we also established a link between the climate conditions and *D. acuminata* interannual variability, it is not clear how the reduction in river streamflow during La Niña conditions ultimately leads to high *D. acuminata* cell densities. Similarly to the observed blooms from their Swedish counterparts, blooms of *D. acuminata* in southern Chile fjords are more frequently reported from permanent salinity-driven stratified systems [39,40], where they show heterogeneous vertical distribution associated with the pycnocline [21,28]. Results from an intertidal experiment in the Patipalena Fjord [28] indicate that the vertical distribution of *D. acuminata* cells is affected by the vertical movement of the pycnocline caused by shear instabilities. Thus, a possibility to be explored is if changes in the streamflow levels affect the microscale circulation features patterns in southern Chilean fjords that could favor the accumulation of cells in the pycnocline.

Another important aspect to be clarified is the origin of the *D. acuminata* populations occurring inside the Reloncaví Fjord. The spatial patterns observed during the 10-year time series (Figure 1F) indicate that the highest cell densities of *D. acuminata* are first observed in areas close to the head of the fjords and posteriorly in the middle portion. Although this suggest that blooms originated in the interior of the fjord and posteriorly transported to the external locations through the surface outflow [80], it is not clear if they are originated from persistent winter populations or advected cells that could enter the fjord through the inflow layer. High *D. acuminata* cell densities correspondent to what is widely considered as bloom level for this species (>1000 cells L^−1^) are occasionally observed during winter conditions (likely remnants from summer blooms). As *Dinophysis* species can survive without prey for months [32,81], these winter cells could constitute suitable inoculum for the next spring–summer populations [34]. Finally, the viability of putative *D. acuminata* overwintering cells (i.e., cells with reddish pigmentation observed at the end of growth season) observed in bottom layers of Reloncaví Fjord [34] should be determined.

The final major question to be answered is if the interannual variability observed in this study was due to factors affecting *D. acuminata* per se or the effect of the environmental conditions on its putative prey. While blooms of *M.* cf. *rubrum* ciliates are a common occurrence in the upwelling areas off Central-Northern Chile [82], the limited quantitative information on the occurrence of *M.* cf. *rubrum* ciliates in southern Chilean fjords and adjacent seas indicates that they are present in low cell densities throughout the year with maximal cell concentrations (and episodic blooms) observed during summer–spring months [21,82,83]. This suggests that the environmental conditions for their development are not usually suitable for mass proliferation within the zone of fjords and channels further south. Of note is the fact that the denser blooms of *M.* cf. *rubrum* ciliates in southern Chile were reported under La Niña conditions, in the years 1975 [84] and 1978 [85] for the Straits of Magellan (54°01′ S; 71°46′ W) and Aysén Fjord (45°22′ S; 73°04′ W), respectively. Thus, one hypothesis to be assessed is if oceanographic conditions during La Niña would facilitate the advection and development of offshore populations in the zone of fjords and channels which would posteriorly result in *Dinophysis* blooms.

### 3.5. Concluding Remarks

The interannual variability in *D. acuminata* and *P. reticulatum* in the Reloncaví Fjord was strongly linked to climatological events of regional scale (i.e., ENSO and SAM), with cold years (La Niña condition) associated with low Puelo River’s streamflow being more favorable to the development of. *D. acuminata* blooms, whereas strong El Niño events coupled to the positive phase of the SAM index lead to *P. reticulatum* blooms. These outcomes become more relevant as anthropogenic climate changes has been reported to cause a tendency in SAM toward its positive phase [52], which could change the current scenario characterizing dinoflagellate blooms in southern Chilean fjords.

## 4. Materials and Methods

### 4.1. Study Area and Datasets

The Reloncaví Fjord (~41.6° S), located in the uppermost region of the Chilean fjord zone, is the site of one of the largest mytilid Chilean production areas (Figure 1). The fjord is 60-km long, has a surface of 170 km^2^, a maximum depth of 460 m and constitutes a representative model for other fjords in the region. The fjord has an annual average streamflow of 650 m^3^ s^−1^ and a pluvio-nival regime. Its circulation is mostly regulated by freshwater input from the Puelo River, which drains a trans-Andean watershed and empties into the middle of Reloncaví Fjord and reaches its maximum streamflow in winter (rainfall) and spring (snowmelt) [43]. Streamflow of the Puelo River is significantly correlated with the streamflow of other rivers that drain into the middle and head of the Reloncaví Fjord as well as with the other main tributary rivers of the coastal systems in western Patagonia [86]. 

Phytoplankton samples were collected from integrated hose-samplers (0–10 m) and immediately fixed with 1% Lugol’s solution. Potentially toxic algae were quantified using an inverted microscope (Olympus CKX41) using sedimentation chambers (20 mL) at 400×, according to Utermöhl [87]. Water temperature (°C), salinity (PSU) and density (σt) profiles were obtained using a Seabird 19 CTD. The Brunt–Väisälä buoyancy frequency (N_BV_, s^−1^) was estimated based on changes of water density over depth [33]. The N_BV_ was estimated for every 1-m interval and the largest value was used as representative of the water column stratification. Monthly accumulated values for streamflow and precipitation data for the hydrological stations Carrera Basilio (41°36′16″ S, 72°12′23″ W) and Puelo (41°39′4″ S, 72°18′42″ W) were obtained from the Climate Explorer [88]. Monthly values for the Niño 3.4 index and the Marshall Southern Annular Mode (SAM) index were obtained from the U.S. National Oceanic and Atmospheric Administration (NOAA) [89]. As the IFOP data series misses information on nutrient concentrations, a complementary analysis was performed using an additional dataset obtained from samples collected every 2–3 weeks from October 2008 and March 2009 from sampling station 8 (published by Alves-de-Souza et al. [21]). CTD (Sea Bird 19-plus) casts were used to obtain real time vertical profiles of salinity, temperature and fluorescence. Guided by the profile reading, five depths were selected: subsurface (1), above (2) and below (3) the pycnocline, the fluorescence maximum (4) and 16 m (5). Besides the variables above mentioned, this dataset includes concentration of NO_3_^−^, PO_4_^3−^ and Si(OH)_4_, water transparency (Secchi disc), as well as the cell densities of the ciliate *M.* cf. *rubrum*. For a detailed description on the sample collection and analyses regarding this dataset, see Alves-de-Souza et al. [21].

### 4.2. Statistical Analysis 

Before the analysis, the 10-year dataset (*n* = 1170) was inspected in order to exclude the sampling dates for which abiotic measurements were not available. Cell densities were previously transformed [ln(x+1)] to reduce the effect of dominant species whereas environmental variables were standardized to values between 0 and 1, based on the minimum and maximum values of each variable [48]. All the statistical analyses described as follows were performed in R software (R Core Team, 2013) using packages freely available on the CRAN repository [90].

#### 4.2.1. Niche Analysis

Data were arranged in one matrix containing the algal cell densities (*Dinophysis* species and *P*. *reticulatum*) and a second matrix containing the environmental variables (i.e., subsurface water temperature, subsurface salinity, Brunt–Väisälä frequency, Niño 3.4 index and SAM index). The OMI analysis [29] was performed using the function *niche* in the ‘ade4’ package [91]. The reasoning behind the OMI analysis was described in detail by Dolédec et al. [29]. Briefly, a PCA was first performed using the environmental matrix to determine the position of the sampling units (SUs) in the multivariate space, with the origin of the PCA axes corresponding to the center of gravity (*G*) of the SUs (i.e., represents the average mean habitat of the sampling domain). Based on the distribution of the species in the different SUs, a center of gravity was calculated for each species considering only the samples where the species occurred. This center of gravity represents the mean habitat condition used by the species. The OMIs for the different species were then estimated by the Euclidean distance between the species center of gravity and *G*. The total inertia is proportional to the average marginality of species and represents a quantification of the influence of the environmental variables on the niche separation of the species [29]. The statistical significance of the calculated marginalities (i.e., OMIs) were tested using Monte Carlo permutations included in the packages ‘ade4’ (10,000 permutations).

#### 4.2.2. Subniche analysis

The WitOMI calculation was performed using the package ‘subniche’ [36] considering the same species and environmental variables mentioned previously for the OMI analysis. The WitOMI is based on parameters similar to the ones calculated in the OMI analysis, but instead of using the entire sampling domain, it considers one subset at time [30]. For the 10-year data series, the subsets were defined a priori (as explained in the Results section), whereas for the complementary WitOMI analysis using the dataset previously published by Alves-de-Souza et al. [21], the subsets were defined by a k-mean cluster analysis of the OMI scores of the SUs using the function *fact* of the package ‘knitr’ [92], with the optimal number of clusters previously determined using the function *fviz_nbclust* of the package ‘factoextra’ [93]. In both cases, the center of gravity of the SUs (*G_k_*) (i.e., mean habitat condition in the subset), and the center of gravity of the different species in the subset (i.e., mean habitat condition used for the species in the subset) were calculated. Based on these parameters, the two additional marginalities (WitOMI*G* and WitOMI*G_k_*) were calculated. For a detailed explanation on the WitOMI analysis, see Karasiewicz et al. [30].

The function *subkrandtest* in the package ‘subniche’ was used to check for differences in the physical and meteorological conditions among the four subsets. The null hypothesis in this test being that “*G_k_* is not different from the overall habitat condition represented by *G*” [94]. The statistical significances of the calculated marginalities (WitOMI*G* and WitOMI*G_k_*) were tested using the function *subnikrandtest* in the ‘subniche’ package. In the case of the WitOMI*G*, the null hypothesis is that “each species within a subset is uninfluenced by its overall average condition” whereas for the WitOMI*Gk* the null hypothesis states that “each species within a subset is uninfluenced by its subset average condition” [94]. Both functions are based on Monte Carlo permutation test (10,000 permutations). A tutorial for the WitOMI analysis is available at [94].

#### 4.2.3. Relevance of Environmental Variables

Correlation among variables was checked by Pearson analysis. The function *envfit* from the package ‘vegan’ [35] was used to fit the environmental variables to the OMI scores. To visualize the frequency of occurrence (based on presence/absence) of *D. acuminata* and *P. reticulatum* related to the different environmental variables, Kernel density estimation (KDE) plots were obtained using the function *geom-density* of the package ‘ggplot2’ [95]. Finally, the relative importance of the different environmental variables to *D. acuminata* and *P. reticulatum* blooms was accessed by conditional inference tree analysis using the function *ctree* in the package ‘party’ [96]. For that, the density of the two species was converted to a categorical variable with two levels: “bloom” and “no bloom”. Bloom levels were stablished as higher than 1000 and 10,000 cells L^−1^ for *D. acuminata* [34] and *P. reticulatum* [97], respectively. Although the conditional inference tree analyses for both species considered all the environmental variables (i.e., water temperature, salinity, Brunt–Väisälä frequency, Niño 3.4 index and SAM index) only the significant variables (*p* < 0.05) associated with blooms were depicted in the trees.

## Supplementary Materials

The following are available online at http://www.mdpi.com/2072-6651/11/1/19/s1. Figure S1: (A–H) Box-plots showing seasonal median values for the physical and meteorological variables in the different years. (I) Examples of salinity vertical profiles related to the different values of the Brunt–Väisälä buoyancy frequency. Figure S2: Salinity vertical profiles. Figure S3: Box-plots showing seasonal median values for *Dinophysis acuminata* (A) and *Protoceratium reticulatum* (B) in the different years. Table S1: Niche parameters estimated through the OMI analysis for the five *Dinophysis* species and *Protoceratium reticulatum* in the Reloncaví Fjord during the 10-year time series. Table S2: Subniche parameters estimated through the WitOMI analysis of *Dinophysis acuminata* (Dacum) and *Protoceratium reticulatum* (Pret) in the Reloncaví Fjord for the summer months during the 10-year time series.
