# Supplementary material for: Interannual Variability of Dinophysis acuminata and Protoceratium reticulatum in a Chilean Fjord: Insights from the Realized Niche Analysis
Source: Toxins (Basel). 2019 Jan 5;11(1):19. doi: 10.3390/toxins11010019 (PMC6356771; doi:10.3390/toxins11010019)
Supplement: Supplementary file 1 [file toxins-11-00019-s001.pdf]

Supplementary Materials: Interannual Variability of *Dinophysis acuminata* and *Protoceratium reticulatum* in a Chilean Fjord: Insights from the Realized Niche Analysis

Catharina Alves-de-Souza, José Luis Iriarte and Jorge I. Mardones

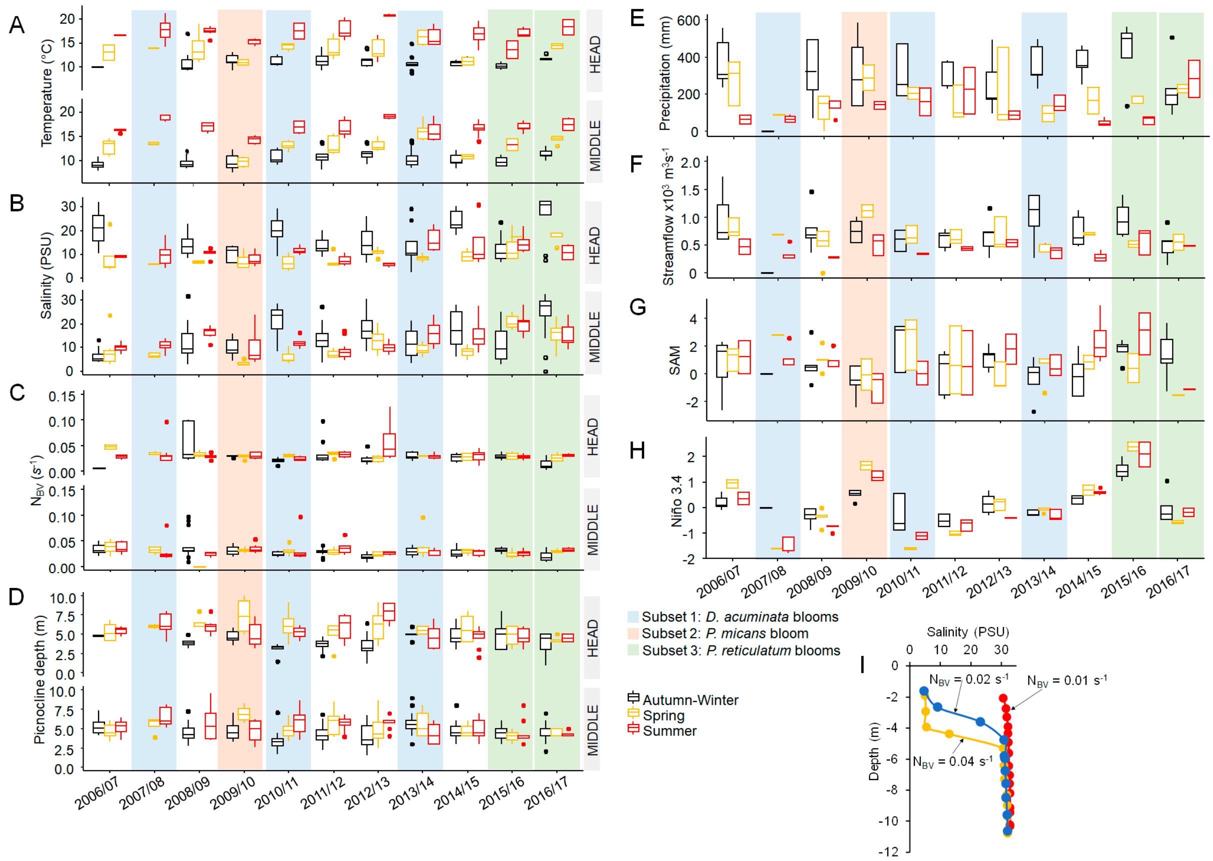

Figure S1. (**A–H**) Boxplots showing seasonal median values for the physical and meteorological variables in the different years. (**I**) Examples of salinity vertical profiles related to the different values of the Brunt-Väisälä buoyancy frequency.

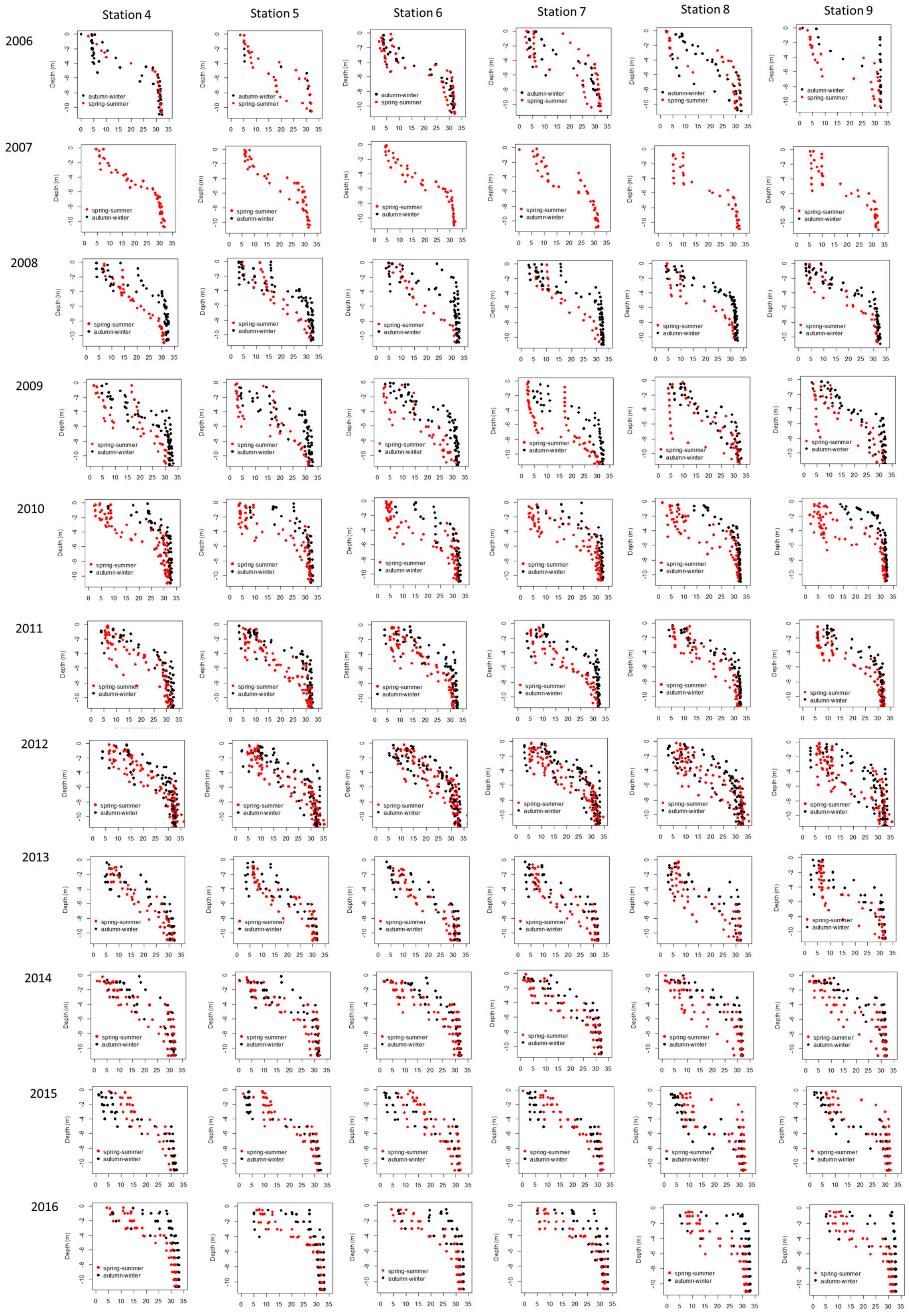

Figure S2. Salinity vertical profiles.

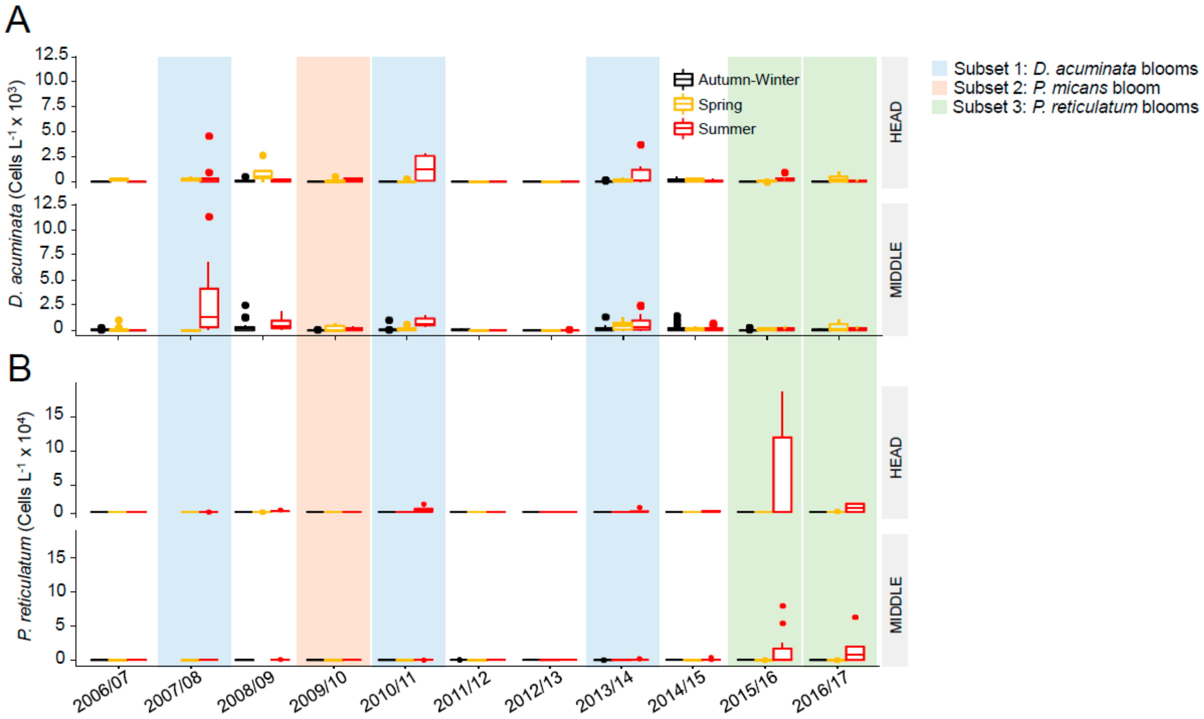

Figure S3. Box-plots showing seasonal median values for *Dinophysis acuminata* (A) and *Protoceratium reticulatum* (B) in the different years.

Table S1. Niche parameters estimated through the OMI analysis for the five *Dinophysis* species and *Protoceratium reticulatum* in the Reloncaví fjord during the 10-years time series. Niche parameters are given both as absolute values and percentages of variability of each species. Inertia = total variability, OMI = outlying mean index (i.e., marginality), Tol = Tolerance, Rtol = Residual Tolerance, OMI% = percentage of variability given by OMI, Tol% = percentage of variability given by Tol, Rtol% = percentage of variability given by Rtol. *P*-values are given by the nu number of random permutations (out of 10,000) that yielded a higher value than the observed marginality (OMI). Species in bold were the ones significant ($p < 0.05$).

**Species**	**Code**	**Inertia**	**Absolute Values**			**Percentages of Variability**			***p*-value**
			OMI	Tol	Rtol	OMI%	Tol%	Rtol%	
D. acuminata	Dacum	12.65	0.61	3.26	8.77	4.80	25.80	69.40	0.009
D. acuta	Dacut	7.36	6.06	0.32	0.98	82.30	4.30	13.40	0.405
D. caudata	Dcau	20.11	18.80	0.01	1.29	93.50	0.10	6.40	0.009
D. tripos	Dtrip	18.66	5.84	3.63	9.19	31.30	19.40	49.30	0.029
D. punctata	Dpunc	9.65	0.80	2.10	6.74	8.40	21.80	69.80	0.158
P. reticulatum	Pret	15.21	5.75	1.07	8.38	37.80	7.10	55.10	0.009

Table S2. Subniche parameters estimated through the WitOMI analysis of *Dinophysis acuminata* (Dacum) and *Protoceratium reticulatum* (Pret) in the Reloncaví fjord for the summer months during the 10-years time series. Subniche parameters are given both as absolute values and percentages of variability of each species. WitOMI*G* = distance between the species center of gravity in the subset and mean habitat conditions (*G*); Tol*G* = Tolerance in the subset related do *G*, RtolG = residual tolerance in the subset related do *G*, OMI*G*% = percentage of variability given by WitOMI*G*, Tol*G*% = percentage of variability given by Tol*G*, Rtol*G*% = percentage of variability given by Rtol*G*, Inertia*G* = total variability for WitOMI*G*. WitOMI*Gk* = distance between the species center of gravity in the subset and mean habitat conditions of the subset (*Gk*); Tol*Gk* = Tolerance in the subset related do *Gk*, RtolG*k* = residual tolerance in the subset related do *Gk*, WitOMI*Gk*% = percentage of variability given by WitOMI*Gk*, Tol*Gk*% = percentage of variability given by Tol*Gk*, Rtol*Gk*% = percentage of variability given by Rtol*Gk*, Inertia*Gk* = total variability for WitOMI*Gk*. All subniches were significative ($p < 0.01$).

Species	Inertia*G*	Absolute Values			Percentages of Variability		
		WitOMI*G*	Tol*G*	Rtol*G*	WitOMI*G*%	Tol*G*%	Rtol*G*%
Subset 1: Summer months of years of *D. acuminata* blooms (2008, 2011 and 2014)							
Dacum1	11.79	1.747	0.420	9.621	14.8	3.6	81.6
Pret1	8.31	3.141	0.246	4.919	37.8	3	59.2
Subset 2: Summer months of the year of *P. micans* bloom (2009)							
Dacum2	15.95	13.574	0.256	2.118	85.1	1.6	13.3
Pret2	12.96	12.347	0.001	0.614	95.3	0	4.7
Subset 3: Summer months of years of *P. reticulatum* blooms (2016 and 2017)							
Dacum3	10.39	6.405	2.235	1.749	61.7	21.5	16.8
Pret3	9.71	3.126	1.083	5.498	32.2	11.2	56.6
Subset 4: Summer months of years with no dinoflagellate blooms							
Dacum4	12.19	10.543	0.174	1.474	86.5	1.4	12.1
Pret4	13.543	5.446	0.476	7.621	40.2	3.5	56.3
Subset 1: Summer months of years of *D. acuminata* blooms (2008, 2011 and 2014)							
Dacum1	10.21	0.17	1.84	8.21	1.60	18.00	80.40
Pret1	7.23	2.07	0.23	4.93	28.60	3.20	68.20
Subset 2: Summer months of the year of *P. micans* bloom (2009)							
Dacum2	5.08	2.71	1.26	1.12	53.30	24.70	22.00
Pret2	2.28	1.66	0.01	0.61	730	0.40	26.60
Subset 3: Summer months of years of *P. reticulatum* blooms (2016 and 2017)							
Dacum3	8.78	4.80	1.25	2.74	54.60	14.20	31.20
Pret3	9.25	2.67	0.21	6.37	28.80	2.30	68.90
Subset 4: Summer months of years with no dinoflagellate blooms							
Dacum4	9.26	7.61	0.12	1.53	82.20	1.30	16.50
Pret4	13.66	5.56	0.86	7.24	40.70	6.30	53.00
